# The Effect of GLT-1 Upregulation on Extracellular Glutamate Dynamics

**DOI:** 10.3389/fncel.2021.661412

**Published:** 2021-03-26

**Authors:** Crystal M. Wilkie, Jessica C. Barron, Kyle J. Brymer, Jocelyn R. Barnes, Firoozeh Nafar, Matthew P. Parsons

**Affiliations:** Division of Biomedical Sciences, Faculty of Medicine, Memorial University, St. John’s, NL, Canada

**Keywords:** glutamate transporter, uptake, ceftriaxone, iGluSnFR, neurotransmission

## Abstract

Pharmacological upregulation of glutamate transporter-1 (GLT-1), commonly achieved using the beta-lactam antibiotic ceftriaxone, represents a promising therapeutic strategy to accelerate glutamate uptake and prevent excitotoxic damage in neurological conditions. While excitotoxicity is indeed implicated in numerous brain diseases, it is typically restricted to select vulnerable brain regions, particularly in early disease stages. In healthy brain tissue, the speed of glutamate uptake is not constant and rather varies in both an activity- and region-dependent manner. Despite the widespread use of ceftriaxone in disease models, very little is known about how such treatments impact functional measures of glutamate uptake in healthy tissue, and whether GLT-1 upregulation can mask the naturally occurring activity-dependent and regional heterogeneities in uptake. Here, we used two different compounds, ceftriaxone and LDN/OSU-0212320 (LDN), to upregulate GLT-1 in healthy wild-type mice. We then used real-time imaging of the glutamate biosensor iGluSnFR to investigate functional consequences of GLT-1 upregulation on activity- and regional-dependent variations in glutamate uptake capacity. We found that while both ceftriaxone and LDN increased GLT-1 expression in multiple brain regions, they did not prevent activity-dependent slowing of glutamate clearance nor did they speed basal clearance rates, even in areas characterized by slow uptake (e.g., striatum). Unexpectedly, ceftriaxone but not LDN decreased glutamate release in the cortex, suggesting that ceftriaxone may alter release properties independent of its effects on GLT-1 expression. In sum, our data demonstrate the complexities of glutamate uptake by showing that GLT-1 expression does not necessarily translate to accelerated uptake. Furthermore, these data suggest that the mechanisms underlying activity- and regional-dependent differences in glutamate dynamics are independent of GLT-1 expression levels.

## Introduction

Ceftriaxone is a cephalosporin antibiotic that is commonly used to enhance glutamate transporter expression in cell and animal models of central nervous system (CNS) disease. As excess glutamate can have detrimental effects on brain tissue (Hardingham and Bading, 2010; Parsons and Raymond, 2014), various excitatory amino acid transporters (EAATs) are required to set both spatial and temporal limits on glutamate’s excitatory actions. In 2005, a screen of over 1,000 compounds demonstrated that ceftriaxone effectively upregulated glutamate transporter-1 (GLT-1) (Rothstein et al., 2005), the brain’s most abundant glutamate transporter. GLT-1 accounts for over 1% of the total tissue protein in the hippocampus (Lehre and Danbolt, 1998). Found primarily on astrocytes—but also present on axon terminals (Chen et al., 2004; Furness et al., 2008)—GLT-1 is an essential glutamate transporter that plays a key role in clearing glutamate following its release into the extracellular space. Knocking out GLT-1 globally (Tanaka et al., 1997) or selectively in astrocytes (Petr et al., 2015) results in lethal seizures. Furthermore, impaired GLT-1 expression and/or function is implicated in a wide variety of CNS conditions. Since ceftriaxone was first demonstrated as a potent stimulator of GLT-1 expression in 2005, it has been used extensively in the literature to provide neuroprotection in disease models. With few exceptions, ceftriaxone shows neuroprotective effects in rodent models of amyotrophic lateral sclerosis, Alzheimer disease, Huntington disease, Parkinson disease, epilepsy and ischemia, to name a few (for recent reviews, see Smaga et al., 2020; Yimer et al., 2019).

The beneficial effects of ceftriaxone in these preclinical studies appear to be straight-forward; ceftriaxone provides neuroprotection by increasing GLT-1 expression and accelerating glutamate uptake, thereby minimizing glutamate toxicity. However, surprisingly few studies have quantified the effect of GLT-1 upregulation on the dynamics of extracellular glutamate following synaptic release. When glutamate uptake is assessed by exposing ceftriaxone-treated cells or tissues to exogenous radiolabeled glutamate over a timescale of minutes, increased absorption of the exogenous glutamate is typically enhanced as a result of the increased GLT-1 protein expression (Rothstein et al., 2005). When provided with 5–10 minutes or more to absorb exogenous glutamate, preparations with more transporters will typically absorb more glutamate. In contrast, synaptically released glutamate transients are extremely fast and can cause a localized increase in the extracellular glutamate concentration to 1 mM for 1–2 milliseconds before returning to basal nanomolar levels (Bergles et al., 1999; Clements et al., 1992). Thus, *in situ* glutamate dynamics are extremely complex and depend on many factors in addition to overall transporter expression levels. For example, glutamate clearance rates depend on the proximity of perisynaptic astrocytic processes to the synapse (Henneberger et al., 2020), synapse size (Herde et al., 2020), astrocyte resting membrane potential (Djukic et al., 2007), transporter surface mobility (Murphy-Royal et al., 2015) as well as the architecture and tortuosity of the extracellular space (Hrabětová, 2005). Posttranslational modifications of glutamate transporters, including phosphorylation and palmitoylation, can also influence transporter-mediated uptake (Casado et al., 1993; Huang et al., 2010; Pita-Almenar et al., 2006). In addition, glutamate clearance is influenced by the duration and frequency of synaptic activity, and can vary in a region-dependent manner (Armbruster et al., 2016; Pinky et al., 2018; Romanos et al., 2019). Despite the widespread use of ceftriaxone to enhance glutamate uptake capacity, it is largely unknown whether pharmacological upregulation of GLT-1 has any influence over the complexities of synaptically released glutamate dynamics in intact tissue.

Here, we used two different compounds—ceftriaxone and LDN/OSU-0212320 (LDN) (Kong et al., 2014)—to increase GLT-1 expression in healthy mice and quantified clearance rates of synaptically released glutamate in real-time using the fluorescence glutamate sensor iGluSnFR (Marvin et al., 2013). We explored the effect of GLT-1 upregulation on glutamate dynamics in multiple brain regions and in response to varying durations of neural activity. We found that despite elevated expression of GLT-1, ceftriaxone and LDN did not speed basal glutamate clearance, had minimal effect on activity-dependent slowing of glutamate clearance, and did not speed glutamate clearance in brain regions characterized by slow glutamate dynamics (e.g. striatum). Furthermore, ceftriaxone decreased glutamate release in the cortex through a GLT-1-independent mechanism. Our results demonstrate the complexities of glutamate uptake and show that increasing GLT-1 expression does not necessarily translate to accelerated uptake.

## Materials and Methods

### Animals

Wild-type (WT) male FVB/N mice were ordered from Charles River at 3–4 weeks of age. They were housed in ventilated cage racks in groups of 3–4 and kept on a 12:12 light:dark cycle with *ad libitum* food and water. All procedures were approved by Memorial University’s Animal Care Committee and were in accordance with the guidelines set by the Canadian Council on Animal Care. After arriving at our housing facility, all mice were allowed a minimum acclimation period of three days before stereotaxic surgery.

### Stereotaxic Surgery

Male FVB/N mice (4–6 weeks of age) were anesthetized by isoflurane inhalation (3%) and maintained with 1.5–2% isoflurane for the duration of the surgical procedure. Mice were secured within the ear bars of a standard stereotaxic apparatus and subcutaneously (s.c.) injected with 0.5 ml of 0.9% sterile saline containing 2 mg/kg meloxicam. A 0.2 ml bolus of 0.2% lidocaine was injected below the scalp and a small incision was then made in the scalp and the underlying skull was exposed. For each region, a total volume of 1 μl of PENN.AAV.GFAP.iGluSnFr.WPRE.SV40 (which was a gift from Loren Looger; Addgene plasmid # 98930; http://n2t.net/addgene:98930; RRID:Addgene_98930) was injected into the cortex, hippocampus or striatum at an injection rate of 5 nl/s. The Hamilton syringe was left in place for an additional 5 minutes following the injection. The following co-ordinates were used with respect to bregma: cortex – 0.7 mm anterior, 2.0 mm lateral, 0.6 mm ventral; hippocampus – 2.6 mm posterior, 2.4 mm lateral, 1.2 to 1.4 mm ventral to brain surface; striatum – 0.7 mm anterior, 2.0 mm lateral, 2.6 mm ventral to brain surface. After the 5 minutes, the syringe was slowly withdrawn, the incision was sutured, and mice were injected with 0.5 ml 0.9% saline (s.c.) before being placed on a heating pad for approximately 30 minutes to accelerate recovery.

### Ceftriaxone and LDN/OSU-212320 Treatments

Following the surgical procedure, mice were injected with either ceftriaxone or LDN. For the ceftriaxone experiments, mice (now 5–7 weeks of age) received intraperitoneal (i.p.) injections of 200 mg/kg/day ceftriaxone for 5–7 days (Rothstein et al., 2005). Ceftriaxone was dissolved in 0.9% saline; thus, control mice received daily i.p. injections of 0.9% saline a day for 5–7 days. Ceftriaxone (or saline) injections began 1–2 weeks after the stereotaxic injection of iGluSnFR. Glutamate imaging experiments and tissue extraction for western blot both occurred the day after the last ceftriaxone injection. For the LDN experiments, mice (now 5–7 weeks of age) received a single i.p. injection of 40 mg/kg (Kong et al., 2014). LDN was dissolved in the vehicle described by Kong et al. (2014); thus, control mice for this group received a single injection of vehicle. The single LDN (or vehicle) injection was administered 2–3 weeks after the stereotaxic injection of iGluSnFR.

### Slice Preparation

The day after the last injection of saline/ceftriaxone, or the day after the single injection of vehicle/LDN, mice were anesthetized with isoflurane, decapitated and the brain was quickly removed and placed in ice-cold oxygenated (95% O^2^/5% CO^2^) slicing solution. Slicing solution consisted of of 125 mM NaCl, 2.5 mM KCl, 25 mM NaHCO_3,_ 1.25 mM NaH_2_PO_4_, 2.5 mM MgCl_2_, 0.5 mM CaCl_2_, 10 mM D-(+)-Glucose. Coronal sections (350 μm) of each brain region were cut using a Leica VT1000 S vibratome. Slices were then recovered at room temperature in oxygenated artificial cerebral spinal fluid (ACSF) composed of 125 mM NaCl, 2.5 mM KCl, 25 mM NaHCO_3,_ 1.25 mM NaH_2_PO_4_, 1.0 mM MgCl_2_, 2.0 mM CaCl_2_, 10 mM D-(+)-Glucose. Slices were left to recover in ACSF for at least 45 minutes before experimentation.

### Imaging and Image Analysis

Slices were transferred to a recording chamber and imaged with an Olympus BX-61 microscope. A peristaltic pump (MP-II, Harvard Apparatus) was used to perfuse oxygenated ACSF at a flow rate of 2 ml/min through the recording chamber throughout the experiments. An in-line heater and temperature controller (TC-344C, Harvard Apparatus) was used to maintain the recording ACSF at a temperature of 32°C. Glass stimulating electrodes (1–2 MΩ resistance) were pulled to a tip resistance of 1–2 MΩ using a Narishige PB-7 pipette puller. Glass stimulating electrodes were filled with ACSF and placed in either the deep layers of the cortex overlying the striatum, the Schaffer collateral pathway of the hippocampus, or in the dorsal striatum. The stimulating electrode was placed at a depth of at least 50 μm below the slice surface. Clampex software (Molecular Devices) was used to coordinate LED illumination (Prior, Lumen 300), electrical stimulation from an Iso-flex stimulus isolator (AMPI), and image acquisition with a high-speed EM-CCD camera (Andor, iXon Ultra 897). iGluSnFR excitation and emission wavelengths were filtered using a standard GFP filter cube and were delivered and collected through a 4×/0.28 NA objective (Olympus). iGluSnFR responses were evoked with 2, 5, 10, 20, 30, 40, or 50 pulses at 100 Hz, delivered at a stimulus intensity of 75 μA. The resulting iGluSnFR transients were recorded using Andor Solis software, with 4 × 4 pixel binning and an exposure time just under 5 ms to achieve an acquisition rate of 205 frames per second. Evoked iGluSnFR responses for 2, 5, 10, 20, 30, 40, and 50 pulses were each averaged over stimulus 5 trials, with non-stimulus trials used to control for mild bleaching. Stimulus and non-stimulus trials were interleaved at an interval of 10 seconds; thus successive stimulation trials were separated by 20 seconds. The average of the non-stimulus trial images were subtracted from the average of the stimulus trials using the IOS and VSD signal processor plugin for ImageJ. Fluorescent intensity changes were quantified in a 10 × 10 pixel (160 × 160 μm) region of interest (ROI) adjacent to the stimulating electrode. These ROIs were drawn 50–100 μm away from the stimulating electrode to avoid areas of tissue damage associated with the electrode placement. Changes in iGluSnFR intensity within the ROI were expressed as %ΔF/F. For each stimulation paradigm (e.g., 2 pulses and 5 pulses), the %ΔF/F values of the iGluSnFR transient were used to calculate a peak response, decay tau and area under the curve (AUC). Decay tau and AUC values were calculated in GraphPad Prism (version 9). Decay tau was calculated by fitting a single-exponential non-linear curve that started at the end of the electrical stimulation. For example, for 50 pulses (100 Hz) starting at time = 0 ms, the curve fit would be applied to time = 500 ms onward. The “fire” heat map in ImageJ was applied to maximal projection stacks to visualize the iGluSnFR response. The “volume viewer” 3D plugin in ImageJ was also used to help visualize the response along the z-(time) axis.

### Western Blotting

Only one hemisphere (right) was injected for iGluSnFR imaging experiments; thus, the non-injected left hemisphere was used to collect tissue for western blot analysis. The cortex, hippocampus, and striatum were each dissected and homogenized, and western blots were performed exactly as described previously (Pinky et al., 2018).

### Drugs

Ceftriaxone was ordered from Sigma-Aldrich (C5793) and dissolved in 0.9% saline solution and administered in a dose of 200 mg/kg. LDN was ordered from Tocris (Cat. No. 5082) and dissolved in a vehicle solution consisting of 500 μl of 1% DMSO/1% polyethylene glycol 400/0.2% Tween 80/10% hydroxypropyl-β-cyclodextrin/saline.

### Statistics

The statistical test used for each analysis is clearly indicated in results text. *p*-values of <0.05 were considered significant. For imaging experiments n-values refer to the number of slices from the following animal numbers: Saline *n* = 7 mice; ceftriaxone *n* = 7 mice; vehicle *n* = 9 mice; LDN *n* = 9 mice.

## Results

### Ceftriaxone Effects on GLT-1 Expression and Glutamate Dynamics in the Hippocampus

**FIGURE 1:**
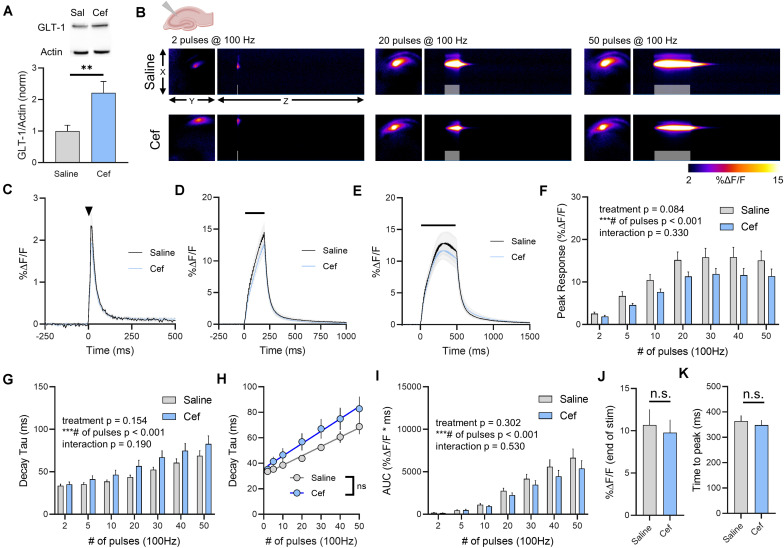
The effect of ceftriaxone on glutamate dynamics in the hippocampus. **(A)** GLT-1 expression in hippocampal tissue from saline and ceftriaxone (Cef)-treated mice. **(B)** Representative images depicting the iGluSnFR response evoked by 2 (left), 20 (middle), and 50 (right) pulses of electrical stimulation at 100 Hz in saline- (top) and Cef-treated (bottom) mice. X–Y images (2048 × 2048 μm) depict the maximal projection image of the iGluSnFR response, while the X–Z image (2048 μm vertically x 1945 ms horizontally) depicts the iGluSnFR response over time (z-axis). Gray shading indicates the onset and duration of electrical stimulation at 100 Hz. **(C–E)** Mean (± S.E.M in gray) iGluSnFR responses to 2 **(C)**, 20 **(D)**, and 50 **(E)** pulses at 100 Hz in saline- and Cef-treated mice. Electrical stimulation is denoted by the arrowhead in **(C)** and the horizontal lines in **(D)** and **(E)**. **(F)** Mean (± S.E.M) iGluSnFR response peaks. **(G)** Mean (± S.E.M) iGluSnFR decay tau values. **(H)** Linear regression to assess the magnitude of the activity-dependent increase in iGluSnFR decay tau. **(I)** Mean (± S.E.M) iGluSnFR area under the curve (AUC). **(J)** Mean (± S.E.M) of the iGluSnFR response size (%ΔF/F) at the termination of the 50-pulse stimulation paradigm (50 pulses at 100 Hz). **(K)** Mean (± S.E.M) of the time required for the iGluSnFR response to reach a peak during the 50-pulse stimulation paradigm. ^∗∗^*p* < 0.01, ^∗∗∗^*p* < 0.001. n.s. not significant. Brain slice schematic in **(B)** was created using Biorender.com.

To explore the effects of GLT-1 upregulation on real-time measures of extracellular glutamate dynamics, healthy male FVB/N mice were treated with ceftriaxone for 5–7 days (200 mg/kg/day, i.p.). Ceftriaxone treatment resulted in a significant increase in total GLT-1 expression in the hippocampus as detected by western blot (Figure 1A, saline *n* = 7, ceftriaxone *n* = 7, *t*-test, *p* = 0.008). This result is in agreement with numerous previous studies on the effects of ceftriaxone on GLT-1 expression in the hippocampus (for review, see Smaga et al., 2020). To determine whether this increased GLT-1 expression had any impact on either basal glutamate clearance or activity-dependent slowing (Armbruster et al., 2016; Pinky et al., 2018) of glutamate clearance in the hippocampus, we visualized extracellular glutamate transients by imaging iGluSnFR at 205 frames per second. Neural activity was evoked by stimulating the Schaffer collateral pathway with a range of stimulations. A heat-map depicting representative iGluSnFR responses to 2, 20, and 50 pulses is shown in Figure 1B, and average iGluSnFR responses to 2, 20, and 50 pulses are shown in Figures 1C–E, respectively. Response peaks, indicative of the relative amount of glutamate released (Koch et al., 2018), were not significantly different in saline compared to ceftriaxone-treated mice, although a strong non-significant trend toward a reduction in iGluSnFR peaks was observed in the ceftriaxone group. As expected, there was a significant effect of the number of pulses, with longer 100 Hz stimulations generating larger iGluSnFR peaks (Figure 1F, saline *n* = 14, ceftriaxone *n* = 16, two-way RM ANOVA, treatment *p* = 0.084, # of pulses *p* < 0.001, interaction *p* = 0.330). Regardless of treatment (i.e., saline or ceftriaxone), the average iGluSnFR response size increased from 2 up to 20 pulses before reaching a plateau.

As ceftriaxone significantly increased hippocampal GLT-1 expression, we asked whether this was reflected by faster iGluSnFR decay tau values, as iGluSnFR decay kinetics have previously been shown to serve as a sensitive measure of relative changes in glutamate clearance rates (Armbruster et al., 2016; Barnes et al., 2020; Dvorzhak et al., 2019; Parsons et al., 2016; Pinky et al., 2018; Romanos et al., 2019). As we and others have shown before (Armbruster et al., 2016; Pinky et al., 2018), increasing the duration of 100 Hz stimulation resulted in activity-dependent slowing of glutamate clearance rates as quantified by the decay tau of the iGluSnFR transient. Unexpectedly, neither basal glutamate clearance nor the activity-dependent slowing of glutamate clearance were affected by ceftriaxone-induced GLT-1 upregulation (Figure 1G, saline *n* = 14, ceftriaxone *n* = 16, two-way RM ANOVA, treatment *p* = 0.154, # of pulses *p* < 0.001, interaction *p* = 0.190). As an additional quantification method to focus exclusively on activity-dependent slowing of glutamate clearance, we used linear regression to determine the relationship between the number of pulses (and therefore, the duration of the 100 Hz stimulation) and the decay tau. We found a significant positive correlation between number of pulses and iGluSnFR decay tau for both saline- (Figure 1H, linear regression, *r* = 0.710, *p* < 0.001) and ceftriaxone-treated mice (Figure 1H, linear regression, *r* = 0.528, *p* < 0.001), further confirming that glutamate clearance is slowed in an activity-dependent manner. However, there was no significant difference between the linear regression slopes for saline- and ceftriaxone-treated mice (*p* = 0.173), suggesting that the activity-dependent slowing of glutamate clearance occurred at similar rates in both groups. In addition, we quantified iGluSnFR AUC as a relative measure of the total amount of extracellular glutamate accumulation during each stimulation. Not surprisingly, iGluSnFR AUC significantly increased with an increasing number of pulses. However, ceftriaxone treatment had no effect on iGluSnFR AUC (Figure 1I, saline *n* = 14, ceftriaxone *n* = 16, two-way RM ANOVA, treatment *p* = 0.302, # of pulses *p* < 0.001, interaction *p* = 0.530). As iGluSnFR responses often peaked prior to the end of the stimulation for the longer stimulus trains, we also quantified the response size at the end of the 50-pulse stimulation, which was not significantly different between saline and ceftriaxone groups (Figure 1J, unpaired *t*-test, *p* = 0.702). Similarly, the time to reach a peak during the 50-pulse stimulation was not affected by ceftriaxone treatment (Figure 1K, unpaired *t*-test, *p* = 0.594). Together, these data demonstrate that while ceftriaxone successfully increased hippocampal GLT-1 expression in the healthy mouse brain, it was without effect on real-time measurements on the extracellular dynamics of synaptically released glutamate. Moreover, GLT-1 overexpression was unable to overcome activity-dependent slowing of glutamate clearance.

### Ceftriaxone Effects on GLT-1 Expression and Glutamate Dynamics in the Cortex

**FIGURE 2:**
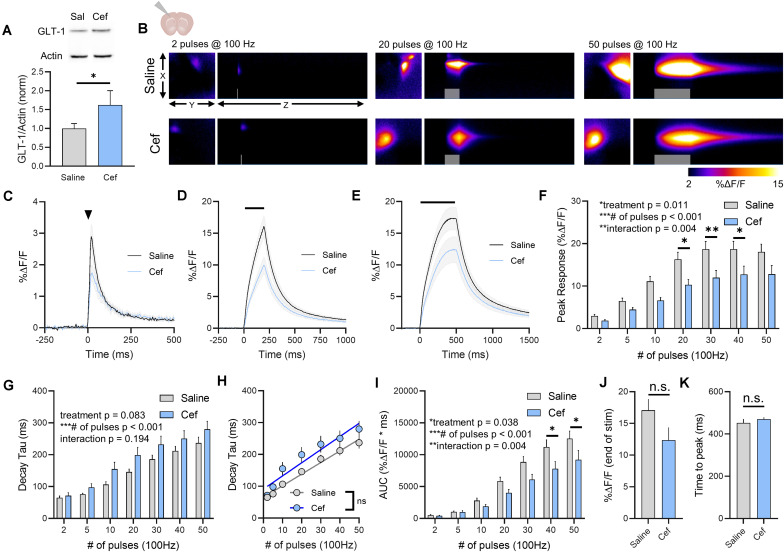
The effect of ceftriaxone on glutamate dynamics in the cortex. **(A)** GLT-1 expression in cortical tissue from saline and ceftriaxone (Cef)-treated mice. **(B)** Representative images depicting the iGluSnFR response evoked by 2 (left), 20 (middle), and 50 (right) pulses of electrical stimulation at 100 Hz in saline- (top) and Cef-treated (bottom) mice. X–Y images (2048 × 2048 μm) depict the maximal projection image of the iGluSnFR response, while the X–Z image (2048 μm vertically x 1945 ms horizontally) depicts the iGluSnFR response over time (z-axis). Gray shading indicates the onset and duration of electrical stimulation at 100 Hz. **(C–E)** Mean (± S.E.M in gray) iGluSnFR responses to 2 **(C)**, 20 **(D)**, and 50 **(E)** pulses at 100 Hz in saline- and Cef-treated mice. Electrical stimulation is denoted by the arrowhead in **(C)** and the horizontal lines in **(D)** and **(E)**. **(F)** Mean (± S.E.M) iGluSnFR response peaks. **(G)** Mean (± S.E.M) iGluSnFR decay tau values. **(H)** Linear regression to assess the magnitude of the activity-dependent increase in iGluSnFR decay tau. **(I)** Mean (± S.E.M) iGluSnFR area under the curve (AUC). **(J)** Mean (± S.E.M) of the iGluSnFR response size (%ΔF/F) at the termination of the 50-pulse stimulation paradigm (50 pulses at 100 Hz). **(K)** Mean (± S.E.M) of the time required for the iGluSnFR response to reach a peak during the 50-pulse stimulation paradigm. Sidak’s multiple comparisons *post hoc* test was used in **(F)** and **(I)**. ^∗^*p* < 0.05, ^∗∗^*p* < 0.01, ^∗∗∗^*p* < 0.001. n.s. not significant. Brain slice schematic in **(B)** was created using Biorender.com.

As glutamate clearance rates differ depending on the brain region under investigation (Pinky et al., 2018), we repeated the above experiments in cortical tissue, specifically the deep layers of the cortex near the border of primary somatosensory and motor cortex. Ceftriaxone treatment resulted in a significant increase in total GLT-1 expression in the cortex as detected by western blot (Figure 2A, saline *n* = 7, ceftriaxone *n* = 7, *t*-test, *p* = 0.045). Neural activity was evoked by stimulating approximately 100–200 μm dorsal to the corpus callosum overlying the striatum. A heat-map depicting representative iGluSnFR responses to 2, 20, and 50 pulses is shown in Figure 2B, and average iGluSnFR responses to 2, 20 and 50 pulses are shown in Figures 2C–E, respectively. Similar to the trend observed in the hippocampus where iGluSnFR peaks tended to be smaller following ceftriaxone treatment, we observed the same decrease here, although the result in the cortex was statistically significant. That is, iGluSnFR response size was reduced in the cortex of ceftriaxone-treated mice compared to saline controls. Response size consistently increased with increased durations of 100 Hz stimulation for both groups, and the response was reduced by ceftriaxone (Figure 2F, saline *n* = 15, ceftriaxone *n* = 16, two-way RM ANOVA, treatment *p* = 0.011, # of pulses *p* < 0.001, interaction *p* = 0.004, with *post hoc* differences observed for 20, 30, and 40 pulses, Sidak’s multiple comparisons test). This result suggests that ceftriaxone has an unexpected effect of decreasing glutamate release in the cortex.

Increasing the duration of 100 Hz stimulation resulted in activity-dependent slowing of glutamate clearance rates as quantified by the decay tau of the cortical iGluSnFR transient. As we saw in the hippocampus, neither basal glutamate clearance nor the activity-dependent slowing of glutamate clearance were affected by ceftriaxone-induced GLT-1 upregulation in the cortex (Figure 2G, saline *n* = 15, ceftriaxone *n* = 16, two-way RM ANOVA, treatment *p* = 0.083, # of pulses *p* < 0.001, interaction *p* = 0.194). In fact, the non-significant trend we observed for a treatment effect (*p* = 0.083) reflected a slight tendency for mean clearance rates to be slower, not faster, following ceftriaxone treatment. Nonetheless, this did not reach statistical significance and therefore we conclude that ceftriaxone was without effect on iGluSnFR decay kinetics in the cortex. There was a significant positive correlation between number of pulses and iGluSnFR decay tau for both saline- (Figure 2H, linear regression, *r* = 0.807, *p* < 0.001) and ceftriaxone-treated mice (Figure 2H, linear regression, *r* = 0.635, *p* < 0.001), confirming that glutamate clearance is slowed in an activity-dependent manner in the cortex. However, there was no significant difference between the linear regression slopes for saline- and ceftriaxone-treated mice (*p* = 0.376), suggesting that the activity-dependent slowing of glutamate clearance occurred at similar rates in both groups. iGluSnFR AUC significantly increased with an increasing number of pulses, and ceftriaxone treatment significantly reduced iGluSnFR AUC (Figure 2I, saline *n* = 15, ceftriaxone *n* = 16, Two-way RM ANOVA, treatment *p* = 0.038, # of pulses *p* < 0.001, interaction p = 0.004, with *post hoc* differences observed for 40 and 50 pulses, Sidak’s multiple comparisons test). iGluSnFR response size at the end of the 50-pulse stimulation was not significantly different between saline and ceftriaxone groups (Figure 2J, unpaired *t*-test, *p* = 0.092). The time to reach a peak during the 50-pulse stimulation was also not affected by ceftriaxone treatment (Figure 2K, unpaired *t*-test, *p* = 0.375). Together, these results demonstrate that ceftriaxone does not accelerate cortical glutamate clearance but can negatively regulate glutamate release during neural activity in the cortex. As we observed in the hippocampus, GLT-1 overexpression was unable to overcome activity-dependent slowing of glutamate clearance in the cortex.

### Ceftriaxone Effects on GLT-1 Expression and Glutamate Dynamics in the Striatum

**FIGURE 3:**
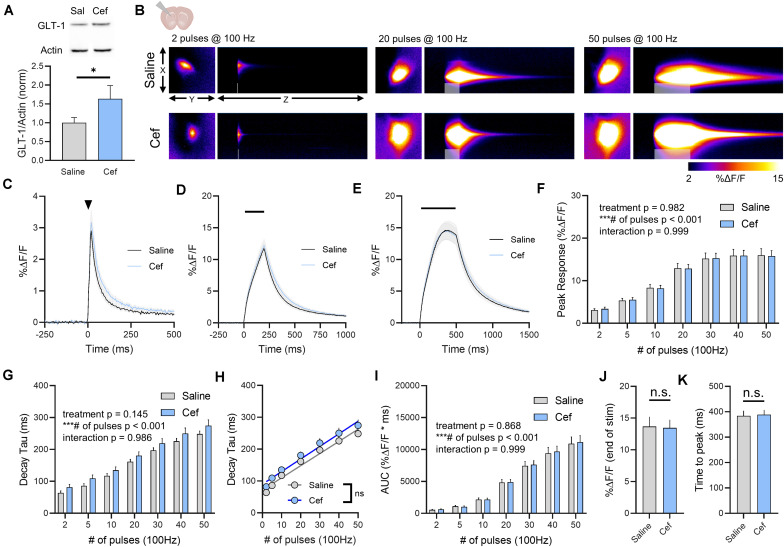
The effect of ceftriaxone on glutamate dynamics in the striatum. **(A)** GLT-1 expression in striatal tissue from saline and ceftriaxone (Cef)-treated mice. **(B)** Representative images depicting the iGluSnFR response evoked by 2 (left), 20 (middle) and 50 (right) pulses of electrical stimulation at 100 Hz in saline- (top) and Cef-treated (bottom) mice. X–Y images (2048 × 2048 μm) depict the maximal projection image of the iGluSnFR response while the X–Z image (2048 μm vertically x 1945 ms horizontally) depicts the iGluSnFR response over time (z-axis). Gray shading indicates the onset and duration of electrical stimulation at 100 Hz. **(C–E)** Mean (± S.E.M in gray) iGluSnFR responses to 2 **(C)**, 20 **(D)**, and 50 **(E)** pulses at 100 Hz in saline- and Cef-treated mice. Electrical stimulation is denoted by the arrowhead in **(C)** and the horizontal lines in **(D)** and **(E)**. **(F)** Mean (± S.E.M) iGluSnFR response peaks. **(G)** Mean (± S.E.M) iGluSnFR decay tau values. **(H)** Linear regression to assess the magnitude of the activity-dependent increase in iGluSnFR decay tau. **(I)** Mean (± S.E.M) iGluSnFR area under the curve (AUC). **(J)** Mean (± S.E.M) of the iGluSnFR response size (%Δ*F*/*F*) at the termination of the 50-pulse stimulation paradigm (50 pulses at 100 Hz). **(K)** Mean (± S.E.M) of the time required for the iGluSnFR response to reach a peak during the 50-pulse stimulation paradigm. ^∗^*p* < 0.05, ^∗∗∗^*p* < 0.001. n.s. not significant. Brain slice schematic in **(B)** was created using Biorender.com.

We repeated the above experiments in the dorsal striatum, as a previous study from our lab demonstrated that glutamate clearance rates in the striatum are particularly slow compared to the hippocampus (Pinky et al., 2018); thus we hypothesized that GLT-1 overexpression may accelerate the slow glutamate clearance characteristic of this area. Ceftriaxone treatment resulted in a significant increase in total GLT-1 expression in the striatum as detected by western blot (Figure 3A, saline *n* = 7, ceftriaxone *n* = 7, *t*-test, *p* = 0.049). Neural activity was evoked by stimulating the dorsal striatum, approximately 100–200 μm ventral to the corpus callosum. A heat-map depicting representative iGluSnFR responses to 2, 20, and 50 pulses is shown in Figure 3B, and average iGluSnFR responses to 2, 20, and 50 pulses are shown in Figures 3C–E, respectively. Unlike the non-significant trend and the significant reduction of iGluSnFR peaks observed in the hippocampus and cortex, ceftriaxone had no effect whatsoever on iGluSnFR peaks in the striatum (Figure 3F, saline *n* = 17, ceftriaxone *n* = 20, two-way RM ANOVA, treatment *p* = 0.982, # of pulses *p* < 0.001, interaction *p* = 0.999). Increasing the duration of 100 Hz stimulation resulted in activity-dependent slowing of glutamate clearance, but there was no effect of ceftriaxone on iGluSnFR decay tau values (Figure 3G, saline *n* = 17, ceftriaxone *n* = 20, two-way RM ANOVA, treatment *p* = 0.145, # of pulses *p* < 0.001, interaction *p* = 0.986).

There was a significant positive correlation between number of pulses and iGluSnFR decay tau for both saline- (Figure 3H, linear regression, *r* = 0.891, *p* < 0.001) and ceftriaxone-treated mice (Figure 3H, linear regression, *r* = 0.748, *p* < 0.001), but there was no significant difference between the linear regression slopes for saline- and ceftriaxone-treated mice (*p* = 0.702), suggesting that the activity-dependent slowing of glutamate clearance occurred at similar rates in both groups. iGluSnFR AUC significantly increased with an increasing number of pulses, but ceftriaxone was without effect on iGluSnFR AUC (Figure 3I, saline *n* = 17, ceftriaxone *n* = 20, two-way RM ANOVA, treatment *p* = 0.868, # of pulses *p* < 0.001, interaction p = 0.999). iGluSnFR response size at the end of the 50-pulse stimulation was not significantly different between saline and ceftriaxone groups (Figure 3J, unpaired *t*-test, *p* = 0.918). The time to reach a peak during the 50-pulse stimulation was also not affected by ceftriaxone treatment (Figure 3K, unpaired *t*-test, *p* = 0.876). In all, evoked iGluSnFR transients were nearly identical between saline- and ceftriaxone-treated mice despite the ceftriaxone-induced increase in GLT-1 expression.

**FIGURE 4:**
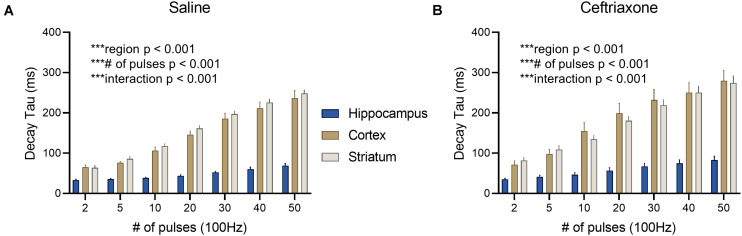
The effect of ceftriaxone on regional differences in glutamate clearance. **(A)** Mean (± S.E.M) iGluSnFR decay tau values to compare regional differences in glutamate clearance in saline-treated mice. **(B)** Mean (± S.E.M) iGluSnFR decay tau values to compare regional differences in glutamate clearance in ceftriaxone-treated mice. ***p < 0.001.

In all, ceftriaxone exhibited no ability to accelerate glutamate clearance rates in either the hippocampus, cortex or striatum. As we demonstrated before, these brain regions exhibit differences in the rate at which they clear evoked glutamate release, with the hippocampus clearing glutamate significantly faster than the cortex and striatum (Pinky et al., 2018). Similar regional differences were observed here (Figure 4A, hippocampus *n* = 14, cortex *n* = 15, striatum *n* = 17, Two-way RM ANOVA, region *p* < 0.001, # of pulses *p* < 0.001, interaction *p* < 0.001), and ceftriaxone did not impact the observed regional differences in clearance rates. That is, regional differences in iGluSnFR decay tau were still readily observed in ceftriaxone-treated mice (Figure 4B, hippocampus *n* = 16, cortex *n* = 16, striatum *n* = 20, two-way RM ANOVA, region *p* < 0.001, # of pulses *p* < 0.001, interaction *p* < 0.001). Together, these data demonstrate that ceftriaxone does not accelerate glutamate clearance in any region tested, and that it can negatively regulate glutamate release in a region-dependent manner.

### LDN/OSU 212320 Effects on GLT-1 Expression and Glutamate Dynamics in the Hippocampus

**FIGURE 5:**
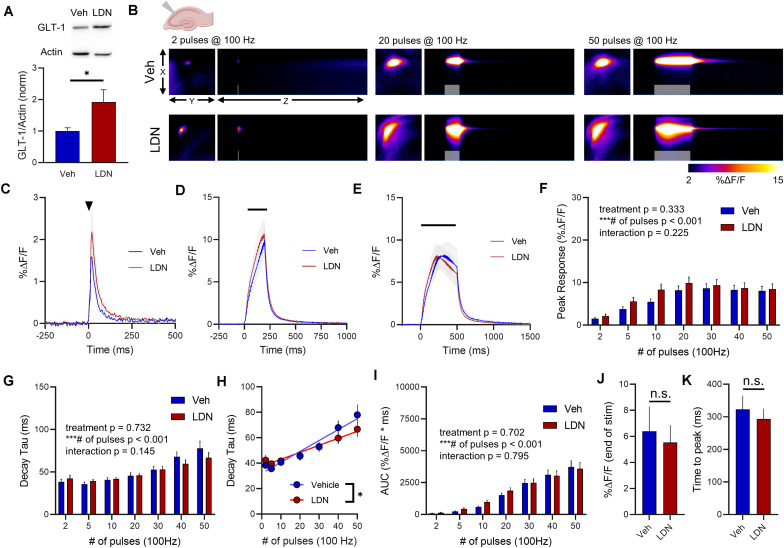
The effect of LDN on glutamate dynamics in the hippocampus. **(A)** GLT-1 expression in hippocampal tissue from vehicle (Veh)- and LDN-treated mice. **(B)** Representative images depicting the iGluSnFR response evoked by 2 (left), 20 (middle), and 50 (right) pulses of electrical stimulation at 100 Hz in Veh- (top) and LDN-treated (bottom) mice. X–Y images (2048 × 2048 μm) depict the maximal projection image of the iGluSnFR response, while the X–Z image (2048 μm vertically x 1945 ms horizontally) depicts the iGluSnFR response over time (z-axis). Gray shading indicates the onset and duration of electrical stimulation at 100 Hz. **(C–E)** Mean (± S.E.M in gray) iGluSnFR responses to 2 **(C)**, 20 **(D)**, and 50 **(E)** pulses at 100 Hz in Veh- and LDN-treated mice. Electrical stimulation is denoted by the arrowhead in **(C)** and the horizontal lines in **(D)** and **(E)**. **(F)** Mean (± S.E.M) iGluSnFR response peaks. **(G)** Mean (± S.E.M) iGluSnFR decay tau values. **(H)** Linear regression to assess the magnitude of the activity-dependent increase in iGluSnFR decay tau. **(I)** Mean (± S.E.M) iGluSnFR area under the curve (AUC). **(J)** Mean (± S.E.M) of the iGluSnFR response size (%ΔF/F) at the termination of the 50-pulse stimulation paradigm (50 pulses at 100 Hz). **(K)** Mean (± S.E.M) of the time required for the iGluSnFR response to reach a peak during the 50-pulse stimulation paradigm. ^∗^*p* < 0.05, ^∗∗∗^*p* < 0.001. n.s. not significant. Brain slice schematic in **(B)** was created using Biorender.com.

More recently, a small molecule called LDN was shown to significantly increase GLT-1 expression (Kong et al., 2014). As LDN likely relies on a different mechanism of GLT-1 upregulation compared to ceftriaxone, we decided to repeat the ceftriaxone experiments, but now comparing LDN treatment to vehicle-treated mice. We reasoned that there may be differences in the subcellular localization and/or functional properties of the GLT-1 produced by LDN treatment compared to ceftriaxone, as we saw no evidence of ceftriaxone to accelerate glutamate clearance rates. LDN treatment resulted in a significant increase in total GLT-1 expression in the hippocampus (Figure 5A, vehicle *n* = 9, LDN *n* = 9, *t*-test, *p* = 0.040). Next, neural activity was evoked by stimulating the Schaffer collateral pathway in the hippocampus. A heat-map depicting representative iGluSnFR responses to 2, 20, and 50 pulses is shown in Figure 5B, and average iGluSnFR responses to 2, 20, and 50 pulses are shown in Figures 5C–E, respectively. LDN did not have any effect on iGluSnFR peaks (Figure 5F, vehicle *n* = 13, LDN *n* = 12, Two-way RM ANOVA, treatment *p* = 0.333, # of pulses p < 0.001, interaction *p* = 0.225) or iGluSnFR decay (Figure 5G, vehicle *n* = 13, LDN *n* = 12, two-way RM ANOVA, treatment *p* = 0.732, # of pulses *p* < 0.001, interaction *p* = 0.145).

There was a significant positive correlation between number of pulses and iGluSnFR decay tau for both saline- (Figure 5H, linear regression, *r* = 0.667, *p* < 0.001) and LDN-treated mice (Figure 5H, *r* = 0.594, linear regression, *p* < 0.001), demonstrating that activity-dependent slowing of glutamate clearance was observed in both groups. However, the slopes of the linear regression lines were significantly different, with LDN-treated mice exhibiting a reduced slope (*p* = 0.021). This result suggests that LDN exerts a mild effect on activity-dependent slowing of glutamate clearance; clearance rates are still slower with increasing durations of activity following LDN treatment, but they do not slow to the same extent as observed in vehicle-treated mice. Total glutamate accumulation was unaffected by LDN treatment, as no significant differences were observed for iGluSnFR AUC (Figure 5I, vehicle *n* = 13, LDN *n* = 12, two-way RM ANOVA, treatment *p* = 0.702, # of pulses *p* < 0.001, interaction *p* = 0.795). iGluSnFR response size at the end of the 50-pulse stimulation was not significantly different between vehicle and LDN groups (Figure 5J, unpaired *t*-test, *p* = 0.722). The time to reach a peak during the 50-pulse stimulation was also not affected by LDN treatment (Figure 5K, unpaired *t*-test, *p* = 0.585).

### LDN/OSU 212320 Effects on GLT-1 Expression and Glutamate Dynamics in the Cortex

**FIGURE 6:**
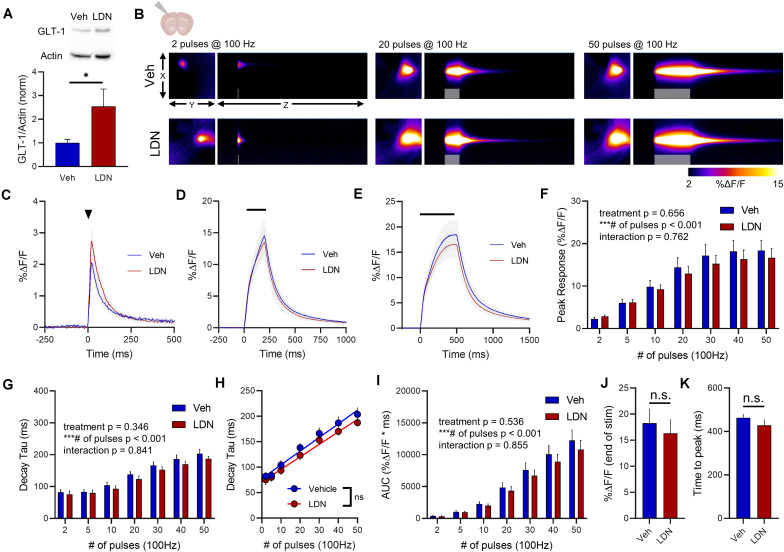
The effect of LDN on glutamate dynamics in the cortex. **(A)** GLT-1 expression in cortical tissue from vehicle (Veh)- and LDN-treated mice. **(B)** Representative images depicting the iGluSnFR response evoked by 2 (left), 20 (middle), and 50 (right) pulses of electrical stimulation at 100 Hz in Veh- (top) and LDN-treated (bottom) mice. X–Y images (2048 × 2048 μm) depict the maximal projection image of the iGluSnFR response while the X–Z image (2048 μm vertically x 1945 ms horizontally) depicts the iGluSnFR response over time (z-axis). Gray shading indicates the onset and duration of electrical stimulation at 100 Hz. **(C–E)** Mean (± S.E.M in gray) iGluSnFR responses to 2 **(C)**, 20 **(D)**, and 50 **(E)** pulses at 100 Hz in Veh- and LDN-treated mice. Electrical stimulation is denoted by the arrowhead in **(C)** and the horizontal lines in **(D)** and **(E)**. **(F)** Mean (± S.E.M) iGluSnFR response peaks. **(G)** Mean (± S.E.M) iGluSnFR decay tau values. **(H)** Linear regression to assess the magnitude of the activity-dependent increase in iGluSnFR decay tau. **(I)** Mean (± S.E.M) iGluSnFR area under the curve (AUC). **(J)** Mean (± S.E.M) of the iGluSnFR response size (%Δ*F*/*F*) at the termination of the 50-pulse stimulation paradigm (50 pulses at 100 Hz). **(K)** Mean (± S.E.M) of the time required for the iGluSnFR response to reach a peak during the 50-pulse stimulation paradigm. ^∗^*p* < 0.05, ^∗∗∗^*p* < 0.001. n.s. not significant. Brain slice schematic in **(B)** was created using Biorender.com.

In the cortex, LDN significantly increased GLT-1 expression compared to vehicle treatment (Figure 6A, vehicle *n* = 9, LDN *n* = 9, *t*-test, *p* = 0.048). A heat-map depicting representative iGluSnFR responses to 2, 20, and 50 pulses is shown in Figure 6B, and average iGluSnFR responses to 2, 20, and 50 pulses are shown in Figures 6C–E, respectively. In the cortex, LDN was without effect on iGluSnFR peak (Figure 6F, vehicle *n* = 10, LDN *n* = 12, two-way RM ANOVA, treatment *p* = 0.656, # of pulses *p* < 0.001, interaction *p* = 0.762) and was also without effect on iGluSnFR decay (Figure 6G, vehicle *n* = 10, LDN *n* = 12, two-way RM ANOVA, treatment *p* = 0.346, # of pulses *p* < 0.001, interaction *p* = 0.841). Both vehicle- and LDN-treated mice exhibited activity-dependent slowing of glutamate clearance (Figure 6H, linear regression, vehicle *r* = 0.815, *p* < 0.001, LDN *r* = 0.803, *p* < 0.001) and the slowing occurred at a similar magnitude (difference between slopes *p* = 0.446). LDN also had no effect on iGluSnFR AUC values in the cortex (Figure 6I, vehicle *n* = 10, LDN *n* = 12, two-way RM ANOVA, treatment *p* = 0.536, # of pulses *p* < 0.001, interaction *p* = 0.855). iGluSnFR response size at the end of the 50-pulse stimulation was not significantly different between vehicle and LDN groups (Figure 6J, unpaired *t*-test, *p* = 0.625). The time to reach a peak during the 50-pulse stimulation was also not affected by LDN treatment (Figure 6K, unpaired *t*-test, *p* = 0.334).

### LDN/OSU 212320 Effects on GLT-1 Expression and Glutamate Dynamics in the Striatum

**FIGURE 7:**
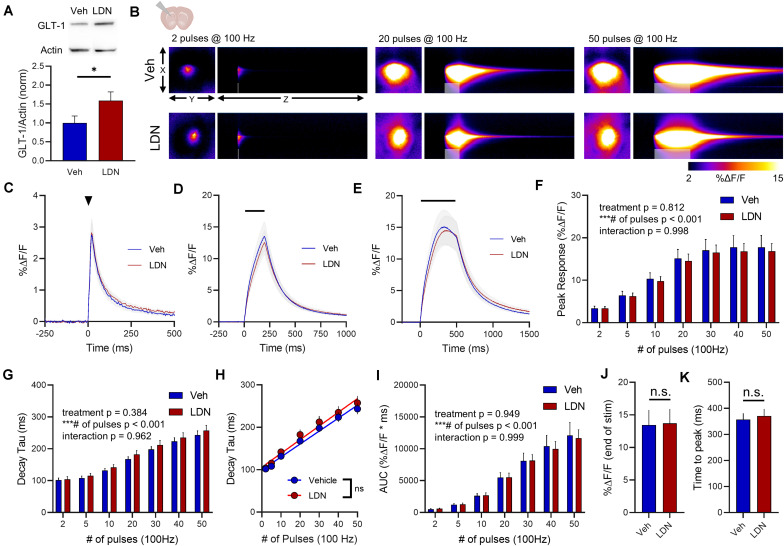
The effect of LDN on glutamate dynamics in the striatum. **(A)** GLT-1 expression in striatal tissue from vehicle (Veh)- and LDN-treated mice. **(B)** Representative images depicting the iGluSnFR response evoked by 2 (left), 20 (middle), and 50 (right) pulses of electrical stimulation at 100 Hz in Veh- (top) and LDN-treated (bottom) mice. X–Y images (2048 × 2048 μm) depict the maximal projection image of the iGluSnFR response, while the X–Z image (2048 μm vertically x 1945 ms horizontally) depicts the iGluSnFR response over time (z-axis). Gray shading indicates the onset and duration of electrical stimulation at 100 Hz. **(C–E)** Mean (± S.E.M in gray) iGluSnFR responses to 2 **(C)**, 20 **(D)**, and 50 **(E)** pulses at 100 Hz in Veh- and LDN-treated mice. Electrical stimulation is denoted by the arrowhead in **(C)** and the horizontal lines in **(D)** and **(E)**. **(F)** Mean (± S.E.M) iGluSnFR response peaks. **(G)** Mean (± S.E.M) iGluSnFR decay tau values. **(H)** Linear regression to assess the magnitude of the activity-dependent increase in iGluSnFR decay tau. **(I)** Mean (± S.E.M) iGluSnFR area under the curve (AUC). **(J)** Mean (± S.E.M) of the iGluSnFR response size (%ΔF/F) at the termination of the 50-pulse stimulation paradigm (50 pulses at 100 Hz). **(K)** Mean (± S.E.M) of the time required for the iGluSnFR response to reach a peak during the 50-pulse stimulation paradigm. ^∗^*p* < 0.05, ^∗∗∗^*p* < 0.001. n.s. not significant. Brain slice schematic in **(B)** was created using Biorender.com.

In the striatum, LDN significantly increased GLT-1 expression compared to vehicle treatment (Figure 7A, vehicle *n* = 9, LDN/OSU *n* = 9, *t*-test, *p* = 0.023). A heat-map depicting representative iGluSnFR responses to 2, 20, and 50 pulses is shown in Figure 7B, and average iGluSnFR responses to 2, 20 and 50 pulses are shown in Figures 7C–E, respectively. In the striatum, LDN was without effect on iGluSnFR peak (Figure 7F, vehicle *n* = 16, LDN *n* = 14, two-way RM ANOVA, treatment *p* = 0.812, # of pulses *p* < 0.001, interaction *p* = 0.998) and was also without effect on iGluSnFR decay (Figure 7G, vehicle *n* = 16, LDN *n* = 14, two-way RM ANOVA, treatment *p* = 0.384, # of pulses *p* < 0.001, interaction *p* = 0.962). Both vehicle- and LDN-treated mice exhibited activity-dependent slowing of glutamate clearance (Figure 7H, linear regression, vehicle *r* = 0.829, *p* < 0.001, LDN *r* = 0.777, *p* < 0.001) and the slowing occurred at a similar magnitude (difference between slopes p = 0.586). LDN also had no effect on iGluSnFR AUC values in the striatum (Figure 7I, vehicle *n* = 16, LDN *n* = 14, two-way RM ANOVA, treatment *p* = 0.949, # of pulses *p* < 0.001, interaction *p* = 0.999). iGluSnFR response size at the end of the 50-pulse stimulation was not significantly different between vehicle and LDN groups (Figure 7J, unpaired *t*-test, *p* = 0.940). The time to reach a peak during the 50-pulse stimulation was also not affected by LDN treatment (Figure 7K, unpaired *t*-test, *p* = 0.709).

**FIGURE 8:**
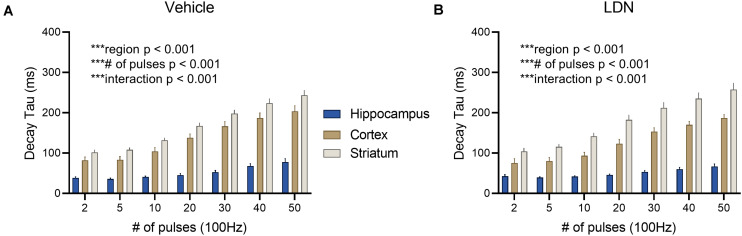
The effect of LDN on regional differences in glutamate clearance. **(A)** Mean (± S.E.M) iGluSnFR decay tau values to compare regional differences in glutamate clearance in vehicle-treated mice. **(B)** Mean (± S.E.M) iGluSnFR decay tau values to compare regional differences in glutamate clearance in LDN-treated mice. ***p < 0.001.

Vehicle-treated mice displayed clear regional differences in glutamate clearance rates (Figure 8A, hippocampus *n* = 13, cortex *n* = 10, striatum *n* = 16, two-way RM ANOVA, region *p* < 0.001, # of pulses *p* < 0.001, interaction *p* < 0.001). Like ceftriaxone, LDN did not accelerate glutamate clearance in the slower regions, and clear regional differences were still observed following LDN treatment despite the increase in GLT-1 expression (Figure 8B, hippocampus *n* = 12, cortex *n* = 12, striatum *n* = 14, two-way RM ANOVA, region *p* < 0.001, # of pulses *p* < 0.001, interaction *p* < 0.001).

## Discussion

In the present study, we demonstrate that pharmacological upregulation of GLT-1 has a minimal impact on real-time measurements of extracellular glutamate dynamics. While our experiments were performed on healthy mice where GLT-1 expression is already expressed at a very high density (Lehre and Danbolt, 1998), we were surprised to find that enhanced GLT-1 expression was generally unable to counter any activity-dependent slowing of glutamate clearance nor was it able to speed glutamate clearance in an area like the striatum, where glutamate clearance is considerably slower than in the hippocampus (Pinky et al., 2018). Using both ceftriaxone and LDN as pharmacological means to increase GLT-1 expression, we found that only LDN was able to exert a small but significant brake on the activity-dependent slowing of glutamate clearance, and this effect was limited to the hippocampus. Both compounds significantly increased total GLT-1 protein expression in the hippocampus, cortex and striatum; yet, aside from the aforementioned LDN effect in the hippocampus, they did not alter basal or activity-dependent changes in glutamate clearance rates. Overall, our data caution the common interpretation that more GLT-1 protein translates to accelerated glutamate uptake. Glutamate uptake is a complex process that relies on much more than just the expression levels of the transporters themselves.

### Comparison With Previous Studies on GLT-1 Upregulation in Healthy Tissue

A recent review paper provides an excellent and thorough review on the effects of ceftriaxone on GLT-1 expression, both in healthy brain tissue and in neurological disease models (Smaga et al., 2020). The authors noted that ceftriaxone consistently increases GLT-1 in the hippocampus, but that ceftriaxone-induced changes in GLT-1 expression were less consistent in other brain regions. In the present study, both ceftriaxone and LDN resulted in a significant increase in GLT-1 expression in the hippocampus, cortex and striatum. Nonetheless, this increased expression was without any clear effect on glutamate uptake rates as measured by evoked iGluSnFR transients in acute brain slices. To complement the summary tables in this recent review paper (Smaga et al., 2020), we have created another table here that summarizes the effects of ceftriaxone on glutamate uptake measurements in healthy (control) cells or tissues (Table 1). In many of these studies, glutamate uptake was quantified by exposing cell cultures, acute slices or synaptosome preparations to exogenous radiolabeled glutamate for approximately 5–10 minutes and measuring how much of the exogenous glutamate was absorbed by the preparation over that time. While serving as an effective means to determine the overall uptake capacity of a given preparation, this technique tells us little about the speed at which glutamate is cleared from the extracellular space following its release from presynaptic terminals. By nature, iGluSnFR decay tau values are significantly slower than the actual uptake rate of glutamate, which is estimated to increase to 1 mM for 1–2 milliseconds following synaptic release (Bergles et al., 1999; Clements et al., 1992); nonetheless, iGluSnFR represents a sensitive means to detect relative changes in clearance rates under different experimental conditions, and can do so on a millisecond timescale. As summarized in Table 1, the bulk of studies that report enhanced glutamate uptake following ceftriaxone treatment quantified the uptake of exogenous radio-labeled glutamate over multiple minutes (Beller et al., 2011; Chotibut et al., 2014; Hu et al., 2015; Lee et al., 2008; Liu et al., 2013; Rothstein et al., 2005; Thöne-Reineke et al., 2008; Verma et al., 2010; Yang et al., 2011). In contrast, the studies that quantified glutamate uptake using iGluSnFR or electrophysiological recordings of transporter currents from astrocytes demonstrate no significant effect of ceftriaxone on glutamate uptake rates in healthy brain tissue (Hefendehl et al., 2016; Rothstein et al., 2005; Wilkie et al., 2020). In the present study, we extend on this observation by showing that GLT-1 upregulation by either ceftriaxone or LDN has very little impact whatsoever on the speed at which synaptically released glutamate is cleared from the extracellular space. Similarly, ceftriaxone treatment in healthy mice does not affect the decay kinetics of NMDAR-mediated excitatory postsynaptic currents (Shen et al., 2014; Valtcheva and Venance, 2016). In addition to cautioning the common interpretation that GLT-1 expression correlates with uptake rates, our data demonstrate that activity-dependent slowing of glutamate clearance is not overcome by GLT-1 upregulation and that the slow clearance (relative to the hippocampus) in the cortex and the striatum is not due to low GLT-1 expression.

**TABLE 1:** Effects of ceftriaxone on glutamate uptake in healthy cells, tissues, and animals.

References	Preparation	Ceftriaxone treatment	Uptake quantification	Effect on uptake
Rothstein et al., 2005	Mouse cortex homogenates	200 mg/kg/day for 7 days (i.p.)	L-[^3^H]-glutamate for 10 min	∼150% increase
Rothstein et al., 2005	Mouse Spinal cord cultures	10–100 μM, applied for 7 days	L-[^3^H]-glutamate for 10 min	∼200% increase (100 μM)
Lee et al., 2008	Primary human fetal astrocytes	10 μM, applied for 2 days	L-[^3^H]-glutamate for 10 min	∼115% increase
Miller et al., 2008	Mouse striatum *in vivo* microdialysis	200 mg/kg/day for 7 days (i.p.)	No-net-flux microdialysis	∼25% increase
Thöne-Reineke et al., 2008	Rat cortical astrocyte cultures	10 μM, applied for 5 days	L-[^3^H]-glutamate for 10 min	∼20% increase
Verma et al., 2010	Rat glial enriched fraction	100 mg/kg/day for 5 days (i.v.)	L-[^3^H]-glutamate for 30 min	∼85% increase
Beller et al., 2011	Rat cortical cultures (mixed neuron/glia)	100 μM and 1 mM, applied for 5 days	L-[^3^H]-glutamate for 10 min	∼30% increase (100 μM); ∼250% increase (1 mM)
Yang et al., 2011	Mouse lumbar spinal cord synaptosomes	200 mg/kg/day for 7 days (i.p.)	L-[^3^H]-glutamate for 10 min	∼125% increase
Liu et al., 2013	Rat primary neuronal cultures	1 μM, applied for 2 days	L-[^3^H]-glutamate for 10 min	∼20% increase
Chotibut et al., 2014	Rat striatal synaptosomes	200 mg/kg/day for 7 days (i.p.)	14C(U)-L-glutamate for 1.5 min	∼50% increase
Hu et al., 2015	Rat hippocampal cell suspension	200 mg/kg/day for 7 days (i.p.)	L-[^3^H]-glutamate for 15 min	∼125% increase
Carbone et al., 2012	Mouse striatal astrocyte cultures	10 μM, 100 μM or 1 mM, applied for 3 days	L-[^3^H]-glutamate for 5 min	No significant effect at 10 μM, 100 μM; ∼15% decrease at 1 mM
Rothstein et al., 2005	Acute hippocampal slices	200 mg/kg/day for 7 days (i.p.)	Transporter currents measured from individual astrocytes	No significant effect
Melzer et al., 2008	Rat mixed glial cultures	10–500 μM, applied for 5 days	L-[^3^H]-glutamate for 5 min	No significant effect
Trantham-Davidson et al., 2012	Rat acute nucleus accumbens core slices	200 mg/kg/day for 5 days (i.p.)	L-[^3^H]-glutamate for 15 min	No significant effect
Zhang et al., 2015	Rat cortical astrocyte cultures	100 μM, applied for 2 days	D-[^3^H]-aspartate for 10 min	No significant effect
Hefendehl et al., 2016	*In vivo* two-photon imaging	200 mg/kg/day for 5 days (i.p.)	Decay of sensory-evoked iGluSnFR transients	No significant effect
Higashimori et al., 2016	Mouse cortical synaptosomes	200 mg/kg/day for 21 days (i.p.)	L-[^3^H]-glutamate for 6 minutes	No significant effect
Agostini et al., 2020	Zebrafish isolated brain tissue	100 μM, 5 × 1 h applications over 3 days	L-[^3^H]-glutamate for 7 min	No significant effect
Wilkie et al., 2020	Mouse acute hippocampal slices	200 mg/kg/day for 7 days (i.p.)	Decay of synaptically evoked iGluSnFR transients	No significant effect

### Ceftriaxone but Not LDN Reduced Glutamate Release in the Cortex

One unexpected observation was that following ceftriaxone treatment, iGluSnFR peaks were significantly reduced in the cortex. This effect in unlikely to be explained by increased GLT-1 expression, as LDN treatment also increased GLT-1 expression but was without effect on iGluSnFR response size. Ceftriaxone has been shown to also increase the activity of the cystine/glutamate antiporter system x_*c*_^–^ (Lewerenz et al., 2009). System x_*c*_^–^ exchanges extracellular cystine for intracellular glutamate, thereby moving glutamate into the extracellular space. The extracellular glutamate derived from the antiporter can increase the endogenous glutamate tone acting on presynaptic group II/III metabotropic glutamate receptors that inhibit canonical synaptic glutamate release (Baker et al., 2002; Baskys and Malenka, 1991; Bridges et al., 2012; Moran et al., 2005). The group II/III metabotropic glutamate receptor agonist LY379268 has previously been shown to reduce evoked iGluSnFR peaks (Koch et al., 2018). While this provides a putative explanation for ceftriaxone’s effect on iGluSnFR peak, it is not clear why such an effect would only be observed in the cortex. Indeed, system x_*c*_^–^ is known to make a considerable contribution to extracellular glutamate levels elsewhere, particularly in the striatum (Baker et al., 2002), and ceftriaxone had no effect whatsoever on iGluSnFR peaks in the striatum in the current study. Alternatively, it is possible that ceftriaxone treatment reduces cortical neuron excitability. Ceftriaxone was previously shown to reduce neuronal excitability in the spinal cord following cervical nerve root injury (Nicholson et al., 2014). If a similar ceftriaxone-induced reduction in excitability occurs in the cortex of healthy mice, it is conceivable that the stimulus trains in the present study resulted in lower cortical neuron excitation and therefore less glutamate release in ceftriaxone-treated animals. At present, the mechanism underlying the observed ceftriaxone-induced reduction in cortical glutamate release is unknown.

### Ceftriaxone and LDN in Neurological Conditions

Despite the general lack of effect of ceftriaxone and LDN on iGluSnFR measurements of glutamate dynamics in the present study, it is undeniable that both compounds can exert a neuroprotective effect in numerous animal models of brain disease. For example, ceftriaxone has been shown to exert a beneficial effect in animal models of Alzheimer disease (Zumkehr et al., 2015), Parkinson disease (Kumar et al., 2016), Huntington disease (Miller et al., 2008), amyotrophic lateral sclerosis (Rothstein et al., 2005), multiple sclerosis (Melzer et al., 2008), traumatic brain injury (Cui et al., 2014) and ischemia (Hu et al., 2015), among others (for recent reviews see Smaga et al., 2020; Yimer et al., 2019). Ceftriaxone’s aforementioned effect on the cystine/glutamate antiporter system x_*c*_^–^ may partially explain some of its protective effects that are independent of GLT-1 expression. Low intracellular cystine can cause glutathione deficiencies and oxidative stress; thus, by increasing intracellular cystine through cystine/glutamate antiporters, ceftriaxone can provide neuroprotection by boosting antioxidant defense. Indeed, it was shown that ceftriaxone increases nuclear levels of the transcription factor Nrf2, which increases the expression of the xCT subunit of the cystine/glutamate antiporter. The ceftriaxone-induced xCT increase correlated with intracellular levels of the antioxidant glutathione, an effect that offered neuroprotection *in vitro* that was independent of glutamate transporter expression (Lewerenz et al., 2009).

LDN also exhibits protective effects in many similar disease models (Kong et al., 2014; Takahashi et al., 2015). The present study was limited to healthy brain tissue, in an effort to understand how GLT-1 upregulation affects glutamate uptake’s natural heterogeneities from one brain region to the next and following different durations of neural activity. In diseases that are characterized by reduced GLT-1 expression and slow uptake, it is likely that a significant proportion of ceftriaxone’s and LDN’s protective effects indeed result from their ability to normalize GLT-1 expression and uptake rates. Indeed, this was nicely demonstrated in an *in vivo* two-photon iGluSnFR imaging study in the APPPS1 mouse model of Alzheimer disease. In brain areas immediately adjacent to amyloid plaques, the authors noted a slower decay of sensory-evoked iGluSnFR transients. After ceftriaxone treatment and restoration of GLT-1 expression adjacent to amyloid plaques, iGluSnFR decay values were indistinguishable from control mice. Interestingly, and in agreement with the results shown here, ceftriaxone treatment in control mice did not accelerate the decay of sensory-evoked iGluSnFR responses (Hefendehl et al., 2016).

## Conclusion

In sum, our data demonstrate that assumptions on the clearance rate of synaptically released glutamate cannot be made based on GLT-1 expression alone. Both ceftriaxone and LDN significantly increased total GLT-1 levels in the hippocampus, cortex and striatum yet had minimal impact on the clearance rate of extracellular glutamate following evoked synaptic release. Transporter-mediated uptake is complex and relies on numerous factors in addition to the total protein level of GLT-1 in a given region. For example, one critical factor is where the ceftriaxone- and LDN-induced GLT-1 is expressed at the subcellular level. Interestingly, it was shown that LDN/OSU-0215111—a derivative of the LDN compound used in the present study—increased GLT-1 expression in subcellular fractions enriched in perisynaptic astrocytic processes, but not in fractions containing cell bodies or synaptosomes (Foster et al., 2018). The results of this paper suggest that LDN/OSU-0215111 initiates local GLT-1 protein synthesis at perisynaptic astrocytic processes. In contrast, an immunogold electron microscopy study demonstrated that ceftriaxone increased GLT-1 in axon terminals but not in perisynaptic astrocytic processes (Capuani et al., 2016). Together with the results of the present paper, it is suggested that increased GLT-1 protein cannot be interpreted to mean accelerated uptake. Future efforts will focus on real-time measures of glutamate clearance in disease states and the effects of pharmacological normalization of glutamate transporters.

## Data Availability Statement

The original contributions presented in the study are included in the article/supplementary material, further inquiries can be directed to the corresponding author/s.

## Ethics Statement

The animal study was reviewed and approved by the Memorial University Institutional Animal Care Committee.

## Author Contributions

CW performed the experiments. CW, MP, and JRB analyzed the data. MP, CW, JCB, and KB wrote the manuscript. MP designed the experiments. MP and FN supervised the project. All authors contributed to the article and approved the submitted version.

## Conflict of Interest

The authors declare that the research was conducted in the absence of any commercial or financial relationships that could be construed as a potential conflict of interest.
